# Influence of Habitual Physical Behavior – Sleeping, Sedentarism, Physical Activity – On Bone Health in Community-Dwelling Older People

**DOI:** 10.3389/fphys.2019.00408

**Published:** 2019-04-15

**Authors:** Gladys Onambele-Pearson, Jorgen Wullems, Conor Doody, Declan Ryan, Christopher Morse, Hans Degens

**Affiliations:** ^1^Department of Exercise and Sport Science, Manchester Metropolitan University, Manchester, United Kingdom; ^2^Department of Rehabilitation Sciences, KU Leuven, Leuven, Belgium; ^3^Faculty of Health and Society, University of Northampton, Northampton, United Kingdom; ^4^School of Healthcare Science, Manchester Metropolitan University, Manchester, United Kingdom; ^5^Institute of Sport Science and Innovations, Lithuanian Sports University, Kaunas, Lithuania; ^6^University of Medicine and Pharmacy of Târgu Mureş, Târgu Mureş, Romania

**Keywords:** accelerometry, aging, bone mineral density, physical behavior, Z-score

## Abstract

Sedentary behavior (SB) has emerged as an independent public-health risk and may contribute to the lower bone mineral density (BMD) in old (>60 years of age) than young adults. The purpose of this study was to quantify SB and habitual physical behavior (PB) in community-dwelling older adults and how this correlates with BMD. In 112 relatively healthy and independent-living individuals aged 72.5 ± 6.4 years, BMD, PB and SB were determined using dual energy X-ray absorptiometry and 7-day three-dimensional accelerometry, respectively. In men, only healthy and osteopenic BMDs were found, whereas in women, osteoporotic BMD classifications also occurred. Our sample spent ∼61%, 7%, 12% and 19% of daily waking hours in SB, standing, LIPA [light intensity physical activity (PA)] and MVPA (medium-to-vigorous intensity PA), respectively. In men, after accounting for covariates (BMI, total fat, android:gynoid ratio), sleeping (hours/day), number of breaks in SB, number of SB ≥ 5 min, number of PA bouts, total duration of PA bouts (min), mean PA bouts duration (min), LIPA (%PA bout time) and MVPA (%PA bout time) were all predictors of BMD. In women, after accounting for covariates (age, BMI, total fat, android:gynoid ratio), SB (hours/day), SB (% waking hours), LIPA (hours/day), LIPA (% waking hours), MVPA (% waking hours) and number of short SB (i.e., <5 min), total time spent in PA (min) significantly correlated with BMD. In conclusion, the PB predictors of bone health in older persons include: night time sleeping duration, number of short bouts of SB, number and duration of bouts of PA relative to total waking hours. While radar graphs of PB patterns for healthy, osteopenic, osteoporotic individuals highlighted significant differences in PB between them, they were not consistent with the expectations from the Mechanostat Theory: i.e., more loading leads to better bone. Rather, our results suggest that a balance of activities must be maintained across the PB spectrum, where certain PB parameters are especially impactful in each sex, supporting the recently coined multifactorial-based variations in the Mechanostat threshold.

## Introduction

In Western societies the average older-adult is highly sedentary and spends up to 80% of their waking time in sedentary behavior (SB) that increases the risk of cardio-metabolic, vascular and musculoskeletal dysfunction (Bey and Hamilton, 2003; Matthews et al., 2008; Proper et al., 2011; Wullems et al., 2016; Ryan et al., 2018a,c). During aging people suffer from a loss of bone mass (Alffram and Bauer, 1962). More than three-million individuals in the United Kingdom suffer from osteoporosis, with €1.2 billion being spent to attend to 200,000 annual fractures sustained to the hip, wrist and spine, and for preventative treatment that only maintains rather than cures this condition (NHS, 2016).

Exercise that includes activities of daily living, such as walking, has been found to be associated with a 30% lower risk of falling and fractures in elderly Caucasian women (>65 years) (Cummings et al., 1995). The reduced fall and fracture risk may not only be attributable to larger muscles, greater strength and better balance, but also to increased bone strength. The Mechanostat theory suggests that bone strength increases in response to increased strain on the bone (Frost, 1987a,b) and walking-associated mechanical loading of the skeleton by transmission of ground-reaction forces (GRFs) through locomotor muscles and respective joints and bones has been suggested to contribute to the reduced fracture risk (Kohrt et al., 1997).

However, recent research suggests that such benefits yielded from regular exercise and physical activity (PA) can be reversed equally or in greater magnitude if individuals are sedentary for the rest of their waking day following engagement in PA. For instance, adults performing ≥150 min/week of moderate to vigorous physical activity (MVPA) often display a detrimental dose–response association with total TV viewing time, typically reversing the effects of PA (Healy et al., 2008). This relationship was also recognized by others (Owen et al., 2010) who stated that adults should adhere to public-health guidelines for PA, but if they sit for prolonged periods of time, no changes to metabolic disease risk-factor profile can be witnessed. Furthermore, if prolonged SB, such as sitting, impairs metabolic functioning, it may be that age-associated bone loss is partially attributable to this lifestyle behavior.

Studies into the effects of SB and bone health have attempted to answer this with the majority of insight and understanding being provided through examining the physiological effects of prolonged bed-rest or studies incorporating zero gravity environments into their experimental design. Reduced gravitational loading of the skeleton is a common characteristic shared amongst all these procedures and contributes to both muscular atrophy and bone loss, in some instances a loss of up to 1% of trabecular bone per week (Mazess and Whedon, 1983; Whedon, 1984). Previous research in healthy young adults showed that the decrease in BMD during bed rest was attenuated with regular lower-limb loading exercise (Zerwekh et al., 1998; Kim et al., 2003). However, any association between less extreme SB (such as simply habitual sitting) and therefore more realistic for everyday persons, and bone health is scarcely studied in the young and even less so in older-age.

Given that unloading leads to a reduction in BMD, one might expect that there is a negative correlation between sleep duration and bone strength, as has indeed been observed (Lucassen et al., 2017). This is, however, equivocal and others have found no relationship between sleep duration and BMD (Niu et al., 2015) or even observed that a short sleep duration was associated with a lower BMD (Fu et al., 2011; Lin et al., 2019). It therefore follows that one of our objectives was to assess the impact of sleep on BMD.

Similarly, it is clear that sex plays a key role in differential bone quality of men compared to women, be they younger or older. In fact it is clear that even where age and genetics background is controlled for (through a study on opposite sex-twins across a large age range), the differences are such that males exhibit up to 21% greater BMD than their female counterparts at most tested sites (Naganathan and Sambrook, 2003; Nieves et al., 2005). Sex must therefore be taken into account in assessing any impact of lifestyle factors on bone health.

The purpose of the present cross-sectional study therefore, was to objectively quantify habitual physical behavior (PB) (i.e., total SB and PA, as well as patterns) using three-dimensional accelerometry to establish associations with bone health. It was hypothesized that (1) individuals engaging more frequently in light-intensity and moderate-vigorous exercise demonstrate better bone health than their less active counterparts; (2) greater SB times is associated with poorer bone health; (3) individuals breaking prolonged bouts of SB more frequently display better bone health. PB was measured over 7 days, using a 3-D accelerometer. Dual-energy X-ray absorptiometry (DEXA) was used to quantify the BMD and soft-tissue.

## Materials and Methods

### Study Sample and Ethics

Participants were recruited by word-of-mouth from a number of national organizations and local clubs in Cheshire, United Kingdom [including the University of the Third Age (U3A), Rotary, Age United Kingdom, local golf clubs]. One hundred and twelve adults (men, *N* = 51/women, *N* = 61) volunteered to participate in this study. All were aged between 57 and 89 years (average ± SD = males 73.6 ± 6.2 years, females 71.6 ± 6.4 years). Participants were excluded if they were: <55 years of age, had any untreated cardiovascular disease (CVD), medication, were diabetic, had any clinically/medication-induced mobility and/or lower limbs strength limitations, had recent (<3 months) injury or surgery, presented decreased mental competence to provide informed consent or understand the study instructions. In addition, data on falls risk [Falls Risk Assessment Tool/FRAT (NICE, 2013)] served as a measure of frailty and those scoring ≥3 (i.e., high fall risk) were excluded. It is also notable that 40% of the sample utilized a primary CVD medication (e.g., statins or warfarin).

Full ethical approval was received through the Manchester Metropolitan University Ethics Committee prior to experimentation. The investigators had completed Ionising Radiation Medical Exposure Regulations (IRMER) training (for studies involving radiation to human participants), and further in-house Risk Assessments were performed that are over and above those stipulated by IRMER, and include daily calibrations, wearing a dosimeter for the regular operator, two room dosimeters in the scanning suite, and logging of radiation dose of each scan. Informed written consent was obtained from each participant.

### Anthropometrics and Body Mass Index (BMI)

Participants’ height and weight were assessed (SECA Beam balance scale, Germany; Woodway PPS 70med Klima, Germany) on arrival at the laboratory in a 10-h overnight fasted state, with the participant unshod and wearing a hospital gown. Dual energy X-ray absorptiometry (Hologic, DEXA Discovery W, Reading, United Kingdom) immediately followed anthropometry and health questionnaire completion, to assess body composition and BMI calculation. Thereto, participants laid supine with palms down, fingers splayed and feet inverted to expose the fibular bone for the 7-min scanning procedure (whole body procedure, EF 8.4 lSv).

### Bone Mineral Density (BMD)

The DXA scan was also used to determine bone phenotype (BMD and content), using OnePass technology to eliminate beam overlap errors and image distortion.

Data was later analyzed using Hologic APEX software (version 3.3) with each region of interest (ROI) carefully demarcated by the same researcher (with an ICC of 0.987). We selected five body segments: spine (average of lumbar and thoracic), pelvis, upper limbs (average of left and right sides), ribs (average of left and right sides) and lower limbs (average of left and right sides). Total bone BMD is also reported.

The T-scores for whole body BMD was used to classify participants as: (1) Normal, T-score < 1.0 SD below normal; (2) Osteopenic, 1 < T-score < 2.5 SD below or (3) Osteoporotic T-score > 2.5 SD below normal (WHO, 1994). These correspond to approximately 10–12%, 20%, and 25% lower values than that in the young healthy 30-year-old, respectively. The T-score indicates how many standard deviations of a sex-matched reference population the BMD of a participant differs from that reference population (here an average healthy 30-year-old man or woman).

Z-scores (i.e., a marker of how bone density compares against the average bone density of an age- and sex-matched group) were calculated using sex and ethnic group specific data from the national health and nutrition examination database (NHANES III).

### Physical Activity (PA) and Sedentary Behavior (SB) Analysis

Sedentary behavior and PA data were collected using commercially available accelerometer hardware and software (GeneActiv Action, Activinsights Ltd., Kimbolton, United Kingdom). Accelerometry outputs were further processed using an in-house developed and validated algorithm based on age-specific activity output cut-off points (Wullems et al., 2017).

Participants were fitted with the accelerometer on their first laboratory visit using two waterproof adhesive patches (3M Tegaderm Transparent Film, Bracknell, United Kingdom). Accelerometers were worn at 50% femur length for 6–7 days post DXA scan. During these 6–7 days participants were asked to carry on with their habitual activities of daily living, exercise and resting habits including bathing, showering and swimming. They were provided with two spare adhesive patches to be applied by themselves on top of the original fittings should the adhesion start to loosen during the course of 6–7 days of monitoring. Overall 58/61 women and 51/51 men returned a complete set of PB data.

Physical behavior was then quantified by normalizing the total wear time during waking hours. SB was classified as sitting or lying or activities incurring a metabolic cost of <1.5 METs (Tremblay et al., 2010). Differing levels of PB were classified as follows:

(1) Sleeping
(2) Quiet standing
(3) Light intensity physical activity (LIPA), whereby activities incurred a metabolic cost of <3 METs.
(4) Moderate-vigorous physical activity (MVPA), whereby activities incurred a metabolic cost of >3 METs (Sasaki et al., 2016).

We have previously cross validated this accelerometer data processing approach using directly measured activities in older persons (Wullems et al., 2017). Here, we quantified 26 common components of PB [for detailed definitions read (Ryan et al., 2018a,b)]:

(1) Nine general PBs: Sleeping (hours/24 h), SB (hours/24 h), standing (hours/24 h), LIPA (hours/24 h), MVPA (hours/24 h), SB (% of waking hours), standing (% of waking hours), LIPA (% of waking hours), MVPA (in % of waking hours).
(2) Six PBs specific to SB amount and accumulation pattern: Breaks in SB (a count), <5 min SB bout (a count), ≥5 min SB bout (a count), mean SB bout length (min), Alfa, W50% (min).
(3) Eleven PBs specific to amount of PA and accumulation pattern: PA bouts (a count), PA bouts (total min), mean PA bout length (min), SB (%PA bout time), standing (%PA bout time), LIPA (%PA bout time), MVPA (%PA bout time), ≥10 min MVPA bouts (in total min), ≥10 min MVPA (a count), Sporadic MVPA (in min), total week ≥ 10 min MVPA (in min).

Sleeping was defined as the overnight period in bed. The time of going to bed and getting out of bed was noted down in a diary by the participants and verified by absence of accelerations in the z-direction during this period.

### Statistical Analyses

All analyses were performed using SPSS Version 24 (IBM, Chicago, IL, United States) whereby all data was checked for Parametricity, with tests of normality (Kolmogorov–Smirnov) being conducted each for men and for women separately.

#### Associations

Bivariate correlations (in men only and women only) were then conducted to assess the influence that PA and SB parameters held over total and site-specific BMD. Results following bivariate correlations are displayed as correlation co-efficient (*r*). Partial correlation controlling for known covariates (see below) that influence BMD then took place with coefficients reported as *R_adj_^n^* depending on previously established bivariate significant associations.

#### Covariates

Particular risk factors for low BMD were identified from previous research including age (Riggs et al., 1982; Manolagas and Jilka, 1995), total fat mass (Harris et al., 1992), and general anthropometry. These data were therefore also collected at study onset. The impact of these potential covariates in our study sample was assessed using Pearson (or Spearman’ Rho for non-normal data) bivariate correlation against bone parameters.

#### Z_PB_-Score Graphic Representation of Group Physical Behavior Patterns

For the graphical representation (Microsoft Excel, Version 2013, Washington, DC, United States) of participants’ habitual PB categorized by their bone health (Z-scores sub-populations), we utilized radar graphs as these are arguably a more comprehensive way to contrast the overall PB of healthy vs. unhealthy bone phenotypes. To compare between the PB of the grouping variables (normal range vs. osteopenia vs. osteoporosis) we used ANOVA (three levels of BMD: <0.75 vs. <0.9 vs. ≥0.9 g/cm^2^) or Kruskal–Wallis tests as appropriate, with follow-up *post hoc* pairwise comparisons (Bonferroni corrected unpaired *t*-tests for the former, or Mann–Whitney tests for the latter) where necessary. Here, PB parameters (total amounts and patterns) were standardized to unit-less quantities into Z-scores to obtain an overall PB picture (whereby PB_Z-score = [mean of group – mean of sample] ÷ standard deviation of sample). Unit weighed score (to compute radar graph areas) assigned a negative sign to PBs linked to unloading bones and a positive sign to those linked to loading of bones sites. The quantitative calculation of the differences between the groups’ areas in the radar graph, (between PB Z_PB_-scores of the bone health groupings) was conducted by computing the *Z_PB_-scores* distance using the NORMDIST function in Microsoft EXCEL. This function returns the distance as a percentage of area.

For all inferential tests, statistical significance was accepted at α ≤ 0.05. In this sample of 61 women, threshold for a β = 0.80 in the correlations, required an explained variance of *r*^2^ = 0.12 (i.e., *r* = 0.346). In this sample of 51 men, threshold for a β = 0.80 in the correlations, required an explained variance of *r*^2^ = 0.14 (i.e., *r* = 0.374).

## Results

### Demographics and BMD

Based on their answers to the health questionnaires during the DEXA scanning procedure, it was ascertained that: 88 participants scored low risk in the FRAT, 87 had no history of major illness, 71 were currently using statins, 102 were non-smokers, 81 had not carried out any resistance exercise in the 6 months preceding the laboratory assessments, 5 regularly consumed dairy products, 18 seldom consumed caffeinated products, 101 did not have rheumatoid arthritis, 98 consumed less than three units of alcohol per day and finally 90 took no calcium/vitamin D supplements.

Participants age, anthropometry and bone health (BMD at five sites and total BMD and Z-score) are detailed in Table 1 separately for men and women. The two sexes were well matched for age and BMI.

**Table 1:** Study population anthropometry and bone characteristics by sex.

	Men	Women
**Age (years)**	**73.6 ± 6.2 ^ND^**	**71.6 ± 6.4^ND^**
Mass (kg)	79.1 ± 11.8^ND^	67.3 ± 13.1^ND^
Height (cm)	173.4 ± 7.6	160.2 ± 5.6^ND^
**BMI (kg/m^2^)**	26.3 ± 3.9	**26.2 ± 5.0^ND^**
Total fat (kg)	24.3 ± 6.7^ND^	28.3 ± 8.9^ND^
Android:gynoid ratio	0.49 ± 0.12^ND^	0.37 ± 0.11^ND^
Ribs	0.81 ± 0.10^ND^	0.65 ± 0.00^ND^
Spine	1.23 ± 0.24^ND^	0.97 ± 0.02^ND^
Pelvis	1.28 ± 0.22	1.15 ± 0.22
Upper_limbs	1.79 ± 0.16^ND^	1.42 ± 0.19
Lower_limbs	2.61 ± 0.34	2.09 ± 0.28
**Total**	**1.32 ± 0.20**	**1.09 ± 0.12^ND^**
**Z_score**	**1.57 ± 1.68**	**0.89 ± 1.11**


*^*ND*^ denotes a normally distributed data set. Data are presented as mean ± SD. Bold numbers indicate significant correlations.*

#### Men Only Sample

In men (total *n* = 51), BMD of the ribs was marginally below the ‘healthy’ classification, suggestive of osteopenia at this site (average left and right ribs BMD: 0.81 ± 0.10g/cm^2^). Notably, however, BMD in all other regions and especially the weight-bearing regions tended to be well within the healthy range. The overall average Z-score in the men varied greatly (Z-score: 1.57 ± 1.68).

#### Women Only Sample

Women (total *n* = 61). Worryingly the average BMD of the ribs was suggestive of osteoporosis (BMD < 0.75g/cm^2^). The rest of the BMD sites inferred healthy bone (BMD > 0.9g/cm^2^). The overall average Z-score in the females (Z-score: 0.89 ± 1.11) was greatly varied across the ‘healthy to osteopenic spectrum’ against age and sex matched population, thereby suggesting there would be factors modulating where any individual older woman’s bone health score would reside.

### Covariates

The covariates analyses against BMD sites can be seen in Tables 2A,B. Significant covariates were confirmed in men thus: BMI for spine, pelvis, upper limbs and lower limbs; total fat mass for the lower limbs; android:gynoid ratio for the upper limbs. In women, covariates included: age for upper limbs and total Z-score; both BMI and fat mass for all sites (i.e., ribs, spine, pelvis, upper limbs, lower limbs, total BMD, Z-score); android:gynoid ratio for pelvis and lower limbs. It was noteworthy in this limited age range (females average of 71.6 ± 6.4 years and men average of 73.6 ± 6.2 years), that age was seldom a covariate either site specific BMD and/or overall Z-score in women and not at all for men.

**Table 2:** Bivariate correlation analysis of age, BMI, and fat as potential covariates for BMD.

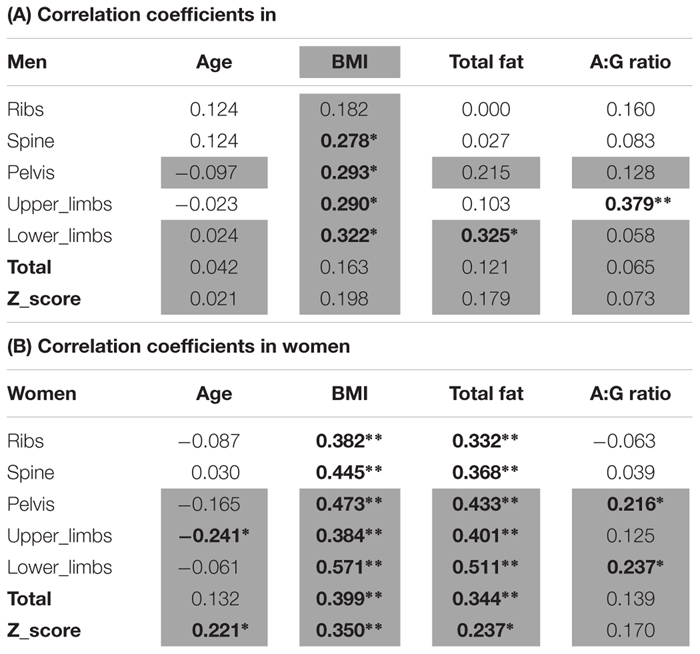

*Analysis was performed between each presumed covariate and specific bone BMD site by sex ^∗^*P* < 0.05; ^∗∗^*P* < 0.01; correlation coefficients are presented. Plain for Pearson and in shaded gray boxes for Spearman’ Rho. **(A)** Correlation coefficients in men. **(B)** Correlation coefficients in women.*

### Physical Behavior in Men and Women

Participants’ habitual physical behaviors or PB (9 general markers, 6 markers related specifically to SB accumulation and pattern, and 11 related specifically to PA accumulation and pattern) are detailed in Table 3.

**Table 3:** Physical behavior of the study population.

		Men	Women
General	Sleeping (hours/24 h)	8.23 ± 0.68^ND^	8.50 ± 0.67
	SB (hours/24 h)	9.68 ± 1.44^ND^	9.44 ± 1.48^ND^
	Standing (hours/24 h)	1.10 ± 0.44^ND^	1.11 ± 0.41^ND^
	LIPA (hours/24 h)	1.91 ± 0.62^ND^	2.05 ± 0.64^ND^
	MVPA (hours/24 h)	3.08 ± 0.89^ND^	2.90 ± 0.86^ND^
	SB (in %/waking hours)	61.44 ± 8.94^ND^	61.03 ± 9.84^ND^
	Standing (%/waking hours)	6.97 ± 2.77^ND^	7.14 ± 2.58^ND^
	LIPA (%/waking hours)	12.10 ± 3.82	13.15 ± 3.96^ND^
	MVPA (%/waking hours)	19.49 ± 5.38^ND^	18.68 ± 5.50^ND^
Sedentary behavior	Breaks in SB (*n*)	22.50 ± 3.80^ND^	22.02 ± 3.33^ND^
	<5 min SB bout (*n*)	6.21 ± 1.83^ND^	6.40 ± 2.14^ND^
	≥5 min SB bout (*n*)	17.06 ± 2.70^ND^	16.41 ± 1.92^ND^
	Mean SB bout length (min)	31.41 ± 9.34	31.29 ± 10.72
	Alfa	1.45 ± 0.04^ND^	1.44 ± 0.04
	W50% (min)	52.92 ± 15.11	53.65 ± 14.19^ND^
Physical activity	PA bouts (*n*)	22.50 ± 3.81^ND^	22.02 ± 3.33^ND^
	PA bouts (total min)	346.75 ± 88.01^ND^	361.75 ± 99.83^ND^
	Mean PA bout length (min)	15.75 ± 4.31^ND^	16.86 ± 5.24^ND^
	SB (%PA bout time)	1.24 ± 0.65	1.25 ± 0.73
	Standing (%PA bout time)	18.10 ± 5.30	18.93 ± 5.35^ND^
	LIPA (%PA bout time)	31.62 ± 4.91^ND^	33.63 ± 5.85^ND^
	MVPA (%PA bout time)	49.04 ± 7.78^ND^	46.20 ± 8.82^ND^
	≥10 min MVPA bouts (total min)	15.16 ± 18.40	11.01 ± 16.32
	≥10 min MVPA (*n*)	0.86 ± 0.85	0.59 ± 0.71
	Sporadic MVPA (min)	162.29 ± 42.14^ND^	166.96 ± 53.29^ND^
	Total week ≥ 10 min MVPA (min)	104.34 ± 126.34	76.45 ± 114.31


*^*ND*^ denotes a normally distributed data set. Data are presented as mean ± SD.*

#### Men Only Sample

During an average day, men spent 8.2 ± 0.7 h sleeping and their waking hours were dominated by SB (61.4 ± 8.9% waking hours), followed by MVPA (19.5 ± 5.4% waking hours) and LIPA (12.1 ± 3.8% waking hours). The average bout length of SB was 31.1 ± 9.2 min, and was longer (*p* < 0.001) than PA (standing, LIPA, MVPA combined) bout length which averaged at 16.0 ± 4.4 min.

#### Women Only Sample

During an average day, women slept slightly more than men with 8.5 ± 0.7 h sleeping. Like men, their waking hours were dominated by SB (61.0 ± 9.8% waking hours), followed by MVPA (18.7 ± 5.5% waking hours) and LIPA (13.2 ± 4.0% waking hours). With SB being the most prevalent behavior, it was also noted that each bout length was 31.1 ± 10.8 min. This SB bout length was longer (*p* < 0.001) than PA (standing, LIPA, MVPA combined) bout length which averaged at 16.6 ± 5.1 min.

### Bivariate Correlations

#### Men Only Correlations

The correlations between BMD and general PB for men can be seen in Table 4A. Sleep time was the only behavior to show any significant association with men’s BMD and this was limited to negative associations with the BMD of the pelvis (*R* = -0.307, *p* < 0.05), upper limbs (*R* = -0.374, *p* < 0.01) and whole body Z-score (*R* = -0.261, *p* < 0.05) indicating that more sleep was associated with lower BMD.

**Table 4:** Bone health and general physical behavior.

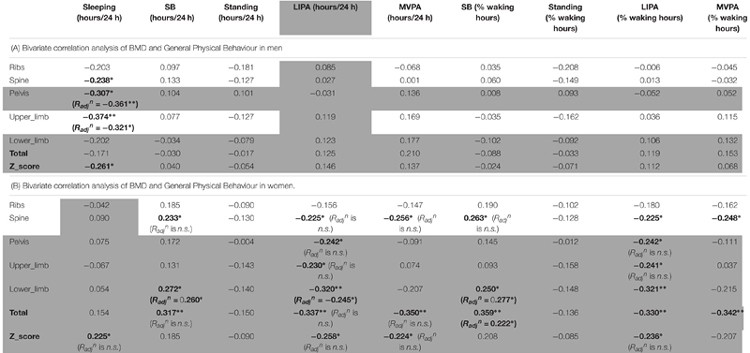

*Analysis was performed between each variable and specific bone BMD site by sex ^∗^*P* < 0.05; ^∗∗^*P* < 0.01 (one-tailed); correlation coefficients are presented. Plain for Pearson and in shaded gray boxes for Spearman’ Rho. *R_*adj*_^*n*^* are the partial correlation results. *n.s.* is non-significant. **(A)** Bivariate correlation analysis of BMD and general physical behavior in men. **(B)** Bivariate correlation analysis of BMD and general physical behavior in women.*

Looking into SB in more details in men (Table 5A), we found that the number of breaks in sedentarism was positively associated with upper limbs BMD (*R* = 0.403, *p* < 0.01), ribs BMD (*R* = 0.282, *p* < 0.05), total BMD (*R* = 0.330, *p* < 0.01) and total Z-score (*R* = 0.296, *p* < 0.05). Interestingly also, the number of short bouts (<5 min) in SB was positively associated with upper limbs BMD (*R* = 0.238, *p* < 0.05). Unexpectedly the number of longer bouts (>5 min) in SB was positively associated with ribs BMD (*R* = 0.349, *p* < 0.01, respectively), lower limbs (*R* = 0.290, *p* < 0.05), total BMD (*R* = 0.373, *p* < 0.01) and total Z-score (*R* = 0.283, *p* < 0.05).

**Table 5:** Bone health and sedentary behavior.

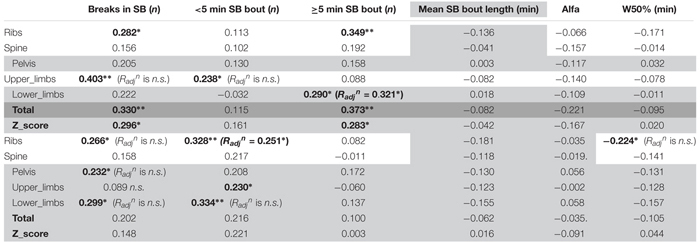

*Analysis was performed between each variable and specific bone BMD site by sex ^∗^*P* < 0.05; ^∗∗^*P* < 0.01 (one-tailed); *n.s.* is non-significant; correlation coefficients are presented. Plain for Pearson and in shaded gray boxes for Spearman’ Rho. *R_*adj*_^*n*^* are the partial correlation results. **(A)** Bivariate correlation analysis of BMD and sedentary behavior in men. **(B)** Bivariate correlation analysis of BMD and sedentary behavior in women.*

As for detailed PA in men (Table 6A), the number of bouts of PA was positively associated with ribs BMD (*R* = 0.282, *p* < 0.05), lower limbs (*R* = 0.238, *p* < 0.05), total BMD (*R* = 0.330, *p* < 0.01) and total Z-score (*R* = 0.296, *p* < 0.05). It was interesting also to note the positive association between MVPA (as a percent of total PA time) and lower limbs BMD (*R* = 0.285, *p* < 0.05). It was surprising that the total duration of PA was only associated, and this negatively, with lower limbs BMD (*R* = -0.254, *p* < 0.05). Equally surprising was that the mean PA bout length was negatively associated with spine BMD (*R* = -0.248, *p* < 0.05), lower limbs BMD (*R* = -0.396, *p* < 0.01), total BMD (*R* = -0.445, *p* < 0.01) and the Z-score (*R* = -0.362, *p* < 0.01). Another unexpected set of results were the negative associations between LIPA and upper limbs BMD (*R* = -0.289, *p* < 0.05), lower limbs BMD (*R* = -0.414, *p* < 0.01) and total BMD (*R* = -0.338, *p* < 0.05). No other associations were significant in the men only sample.

**Table 6:** Bone health and physical activity.

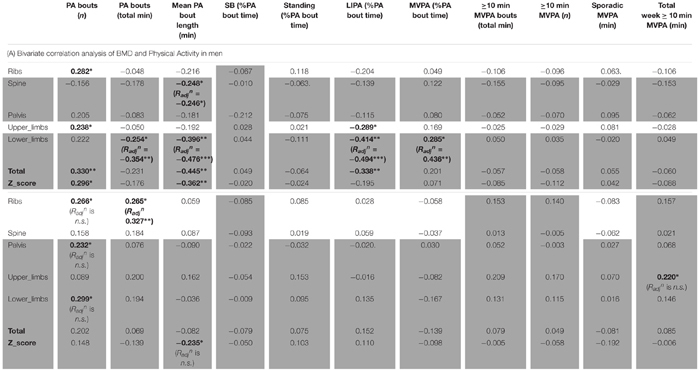

*Analysis was performed between each variable and specific bone BMD site by sex ^∗^*P* < 0.05; ^∗∗^*P* < 0.01; ^∗∗∗^*P* < 0.001 (one-tailed); *n.s.* is non-significant; correlation coefficients are presented. Plain for Pearson and in shaded gray boxes for Spearman’ Rho. *R_*adj*_^*n*^* are the partial correlation results. **(A)** Bivariate correlation analysis of BMD and physical activity in men. **(B)** Bivariate correlation analysis of BMD and physical activity in women.*

#### Women Only Correlations

Women only correlations between BMD and general PB can be seen in Table 4B. Sleep time was in fact positively associated with whole body Z-score (*R* = 0.225, *p* < 0.05). Unexpectedly, SB (in total hours per day) was positively associated with spine BMD (*R* = 0.233, *p* < 0.05), lower limbs BMD (*R* = 0.272, *p* < 0.05) and total BMD (*R* = 0.317, *p* < 0.01). Unexpectedly also, LIPA was negatively associated with all but one site including spine BMD (*R* = -0.225, *p* < 0.05), pelvis BMD (*R* = -0.242, *p* < 0.05), upper limbs BMD (*R* = -0.230, *p* < 0.05), lower limbs BMD (*R* = -0.320, *p* < 0.01), total BMD (*R* = -0.337, *p* < 0.01), and total Z-score (*R* = -0.258, *p* < 0.05). A similar pattern was seen with SB amount expressed as a percent of waking hours. Also unexpected were the negative associations between average daily hours spent in MVPA against spine BMD (*R* = -0.256, *p* < 0.05), total BMD (*R* = -0.350, *p* < 0.01) and total Z-score (*R* = -0.224, *p* < 0.05). A similar pattern was seen with MVPA amount expressed as a percent of waking hours (though not with the Z-score). No other correlations with general PB were significant in the women only sample.

Looking into SB in more details in women (Table 5B), we found that the number of breaks in sedentarism was positively associated with ribs BMD (*R* = 0.266, *p* < 0.05), pelvis BMD (*R* = 0.232, *p* < 0.05) and lower limbs BMD (*R* = 0.299, *p* < 0.05). We also found that the count of short duration (≤5 min) sedentary activities was positively associated with ribs BMD (*R* = 0.328, *p* < 0.01), upper limbs BMD (*R* = 0.230, *p* < 0.05) and lower limbs BMD (*R* = 0.334, *p* < 0.01). Finally, W50% (i.e., the bout duration below which half of all sedentary time is accrued) was negatively associated with ribs BMD (*R* = -0.224, *p* < 0.05).

As for detailed PA in women (Table 6B), this showed very few bivariate associations with bone health. Thus, the number of bouts of PA was positively associated with ribs BMD (*R* = 0.266, *p* < 0.05), pelvis BMD (*R* = 0.232, *p* < 0.05) and lower limbs (*R* = 0.299, *p* < 0.05). Total duration of PA was positively associated with ribs BMD (*R* = 0.265, *p* < 0.05). Mean PA bout length was negatively associated with Z-score (*R* = -0.235, *p* < 0.05). Finally the total weekly accumulation of long (≥10 min) bouts of MVPA was positively associated with upper limbs BMD (*R* = 0.220, *p* < 0.05).

### Partial Correlations

In relevant cases above with significant bivariate correlations, known covariates were then factored into the association using partial correlations, resulting into adjusted correlation coefficient (*R_adj_^n^)*.

#### Men Only Partial Correlations

In men, when adjusting for covariates (i.e., BMI and A:G ratio) the partial associations between upper limbs BMD and short duration SB bouts count, and breaks in SB were nullified (*p* > 0.05) however the correlation against sleep remained (*R_adj_^n^* = -0.321, *p* = 0.012). When adjusting for covariates (i.e., BMI and total fat), the association between lower limbs BMD against many PB parameter was strengthened including MVPA in % PA bout time (*R_adj_^n^* = 0.436, *p* = 0.001), LIPA in % PA bout time (*R_adj_^n^* = -0.494, *p* < 0.001), mean PA bout length (*R_adj_^n^* = -0.476, *p* < 0.001), PA bouts length in min (*R_adj_^n^* = -0.354, *p* = 0.006), long SB bouts count (*R_adj_^n^* = 0.321, *p* = 0.012). Together, this suggests that the associations with upper and lower limbs BMD and many PBS parameters were not just a product of anthropometric factors. Next, the partial correlation between pelvis vs. sleep, controlling for BMI still maintained a significant impact of this PB (*R_adj_^n^* = -0.361, *p* = 0.005). Finally, the partial correlation between spine vs. mean PA bout length, controlling for BMI still maintained a significant impact of this PB (*R_adj_^n^* = -0.246, *p* = 0.042).

#### Women Only Partial Correlations

In women, when adjusting for covariates (age, BMI and total fat), correlations between upper limb BMD and LIPA in % waking hours, number of short SB, and total weekly time in prolonged MVPA were all nullified (*p* > 0.05).

With lower limbs BMD, adjusting for covariates (i.e., BMI, total Fat and A:G ratio), the associations (adjusted correlation coefficient or *R_adj_^n^*) against SB (in hours per day; *R_adj_^n^* = 0.260, *p* = 0.028, and as a percent of waking hours; *R_adj_^n^* = 0.277, *p* = 0.020) and against LIPA in hours per day (*R_adj_^n^* = -0.245, *p* = 0.028) all remained. However, the associations between lower limb BMD and the previously significantly associated number of breaks in SB, number of short SB, number of PA bouts, all disappeared (*p* > 0.05).

With pelvic BMD adjusting for covariates (i.e., BMI, total fat and A:G ratio) partial correlations against LIPA (in hours per day, and in % waking hours), breaks in SB and number of PA bouts all disappeared. The same applied for the spine when adjusting the observed correlations for covariates (i.e., BMI and total fat). This disappearance of correlations between measures of PB patterns with BMD points to the importance of anthropometry and/or body composition in women for the BMD in these sites.

For the ribs the association between BMD with number of short bouts of SB (*R_adj_^n^* = 0.251, *p* = 0.031) and total weekly duration in PA (*R_adj_^n^* = 0.327, *p* = 0.007) remained after adjusting for covariates (i.e., BMI and total fat). However, the partial correlations between rib BMD and number of breaks in SB, W50% and number of PA bouts had disappeared.

For total BMD the partial correlations against SB in % waking hours (*R_adj_^n^* = 0.222, *p* = 0.050) remained after adjusting for covariates (i.e., BMI and total fat), but the associations against SB in hours per day, LIPA in hours per day, and MVPA in hours per day all disappeared.

Finally, the partial correlations of total BMD Z-score against sleep, LIPA in hours per day, LIPA in % waking hours, MVPA in hours per day and mean PA bout length became all statistically non-significant after adjusting for covariates (i.e., age, BMI and total fat).

### Z_PB_ Score Graphic Synthesis of Physical Behavior Patterns by Bone Health Clinical Sub-Groups

None of the parameters of PB consistently correlated with BMD. To evaluate whether PB parameters differed dependent on bone health we classified people as having healthy, osteopenic, and osteoporotic bones, by the T-scores (see methods). We expressed all 26 PB parameters as dimensionless Z_PB_-scores and drew radar graphs to determine whether any patterns in PA were associated with bone health status. This analysis was carried out on lower limbs BMD and upper limbs BMD, separately for men and women.

In the men, the T-score data results revealed none as osteoporotic, 4 men as osteopenic and 47 men as having a healthy skeleton. In the upper limbs this translated to BMDs of 0.77 ± 0.05 g/cm^2^ and 0.90 ± 0.07 g/cm^2^ in the osteopenic and healthy group (*p* < 0.001), respectively, and in the lower limbs BMDs of 1.09 ± 0.07 g/cm^2^ and 1.32 ± 0.16 g/cm^2^ (*p* = 0.001).

**FIGURE 1:**
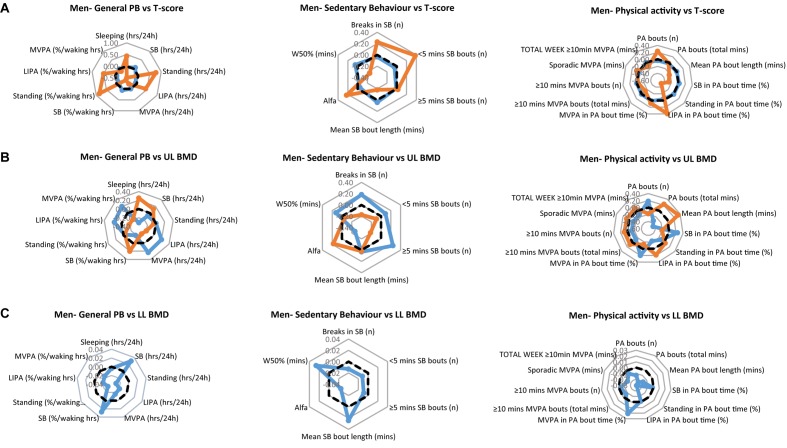
Physical behaviors (PB) in healthy (blue) and osteopenic (orange) men **(A)** as defined by the whole body T-score, **(B)** upper limbs BMD T-score, and **(C)** lower limbs BMD T-score. The radar graphs depict in separate panels (from left to right), general physical behaviors, sedentarism-specific behaviors, and physical activity-specific behaviors. Sample mean is shown in a black dashed line. PB data shown are computed Z-scores.

The PB of each of the men’s clinical groups are illustrated in Figure 1A for T-scores, Figure 1B for upper limbs BMD and Figure 1C for lower limbs BMD. Each figure provides a profile for general PB, SB specific PB and PA specific PB. We especially take note of the lower limbs, since these arguably are the bone sites most likely to be impacted upon by the tracked PB. Here, the men with healthy bones tended to show higher values for SB (hours/day and as % of waking hours) compared to the osteopenic group. However, the healthy lower limb bones on men were associated with significantly lower Standing (hours/day and as % waking hours), significantly lower LIPA (hours/day and as % waking hours) and significantly lower MVPA (hours/day and as % waking hours). Healthy lower limbs BMD were also associated with greater W50% and mean SB bout length (min). More in line with our expectations, however, healthy lower limbs BMD was associated with greater MVPA amount (as % PA bout time).

In the women, the T-score data results revealed 4 women as osteoporotic, 16 women as osteopenic and 40 women as having a healthy skeleton. In their upper limbs this translated to BMDs: osteoporotic: 0.61 ± 0.03 g/cm^2^; osteopenic: 0.66 ± 0.04 g/cm^2^; healthy: 0.74 ± 0.10 g/cm^2^ (all comparisons *p* < 0.001). In their lower limbs this translated to BMDs: osteoporotic: 0.86 ± 0.04 g/cm^2^; osteopenic: 0.93 ± 0.08 g/cm^2^; healthy 1.13 ± 0.17 g/cm^2^ (all comparisons *p* = 0.001).

**FIGURE 2:**
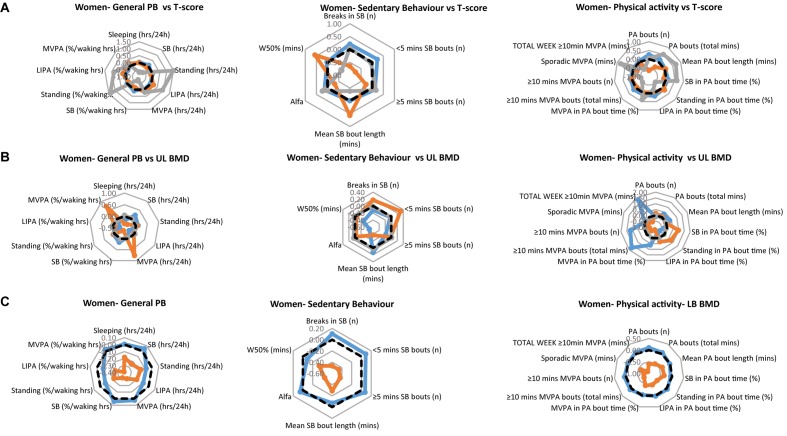
Physical behaviors (PB) in healthy (blue), osteopenic (orange), and osteoporotic (gray) women **(A)** as defined by the whole body T-score, **(B)** upper limbs BMD T-score, and **(C)** lower limbs BMD T-score. The radar graphs depict in separate panels (from left to right), general physical behaviors, sedentarism-specific behaviors, and physical activity-specific behaviors. Sample mean is shown in a black dashed line. PB data shown are computed Z-scores.

The PB of each of the women’s clinical groups, are illustrated in Figure 2A for T-scores, Figure 2B for upper limbs BMD and Figure 2C for lower limbs BMD. Each figure provides a profile for general PB, SB specific PB and PA specific PB. As with the men’s data, we especially take note of the lower limbs. Here, the women with healthy bones tended to show higher values for all general PBs compared to the osteopenic group (there was no osteoporotic lower limbs group in the women sample). Interestingly, the same was also true for all SBs as well as PA behaviors.

In addition, it should be noted that in the women as in the men, the differences in PB between groups tended to be within 1 standard deviation, demonstrating that in absolute terms, the groups had very similar PBs in many cases.

In men, there were no statistical significant differences in general PBs (*P* > 0.05), nor in SB specific parameters (*P* > 0.05) between those with healthy and those with osteopenic bones. However, SB in % PA bout time was less (*p* = 0.037), MVPA in % PA bout time was more (*p* = 0.031) in osteopenic than in normal bone men (Figure 1A, right panel).

In women, there was a main effect of group for standing [in hours per day (*p* = 0.016), and in % waking hours (*p* = 0.022)], LIPA (in hours per day; *p* = 0.037), number of breaks in SB (*p* = 0.010), number of short SB bouts (*p* = 0.002), W50% (*p* = 0.026) and number of PA bouts (*p* = 0.010). This was reflected by longer standing, LIPA (Figure 2A, left panel), and larger number of PA bouts (Figure 2A, right panel) in the osteoporotic than osteopenic and healthy women. The number of breaks of SB was larger and the number of SB bouts smaller in osteoporotic and healthy than osteopenic women, while the W50% was largest in the osteopenic and smallest in the osteoporotic women (Figure 2A, middle panel).

## Discussion

The current study quantified habitual PB (i.e., total SB and PA, as well as patterns) to establish any association with bone health. It was hypothesized that (1) individuals engaging more frequently in light-intensity and moderate-vigorous exercise demonstrate better bone health than their less active counterparts; (2) greater SB time is associated with poorer bone health; (3) individuals breaking prolonged bouts of SB more frequently display better bone health.

The main observation of the present study is that in men out of the possible 182 correlations, 12 supported our hypotheses, 12 went against expectations and the rest (i.e., 158) showed no association. In women, one correlation appeared to support our hypotheses, 12 went against face value expectations and the rest (i.e., 169) showed no association. There were also no expected differences in PB between people with osteoporotic, osteopenic or healthy bones. These observations thus suggest that in contrast to observations of bone loss during bed rest, even SB in older people does not aggravate the aging-related bone loss.

Comparing between T-score categories in men, it transpired that only SB in % PA bout time, and MVPA in % PA bout time differed between healthy vs. osteopenic men. Comparing the PBs of osteoporotic vs. osteopenic vs. healthy T-scores women, revealed group differences in standing (in hours per day, and in % waking hours), in LIPA (in hours per day), in the number of breaks in SB, in the number of short SB bouts, in W50% and in the number of PA bouts.

Bone homeostasis has been demonstrated to become compromised due to a significant age-decline in Vitamin D (>60 years of age), through reduced dietary intake, and decreased exposure to sunlight attributed to mentioned increases in SB (Riggs et al., 1982; Lips, 2001) and indoor activities. Subsequent effects include an over secretion of the parathyroid hormone (hyperparathyroidism), a proven contributor to the loss of cortical bone, causing calcium to be released from a number of reservoirs within both bone and kidney and further depleted and excreted after renal filtration (Riggs et al., 1982; Lips, 2001). In terms of lifestyle, previous research suggests that prolonged bed rest leads to decreased BMD, alongside a prevalence of biochemical markers of bone resorption (NTx), and increased urinary calcium, all factors linked to a causal relationship between PB and bone characteristics.

### Physical Activity and Bone Health

As described above, in contrast to our expectation the level of PA had a negligible effect on BMD. If anything there was a sex bias whereby men tended to show positive links with PA whereas surprisingly, there tended to be a negative association between several BMD data and PA in women.

Total PA for both sexes, and especially MVPA in men and to a lesser extent in women, were in fact significant predictors of bone health. Our findings, even in this group that seldom engaged in MVPA (19.49 ± 5.38% vs. 18.68 ± 5.50% of waking hours, respectively, in men and in women) are in agreement with previous studies reporting greater femoral BMD following engagement in habitual daily physical activities such as walking and stair climbing (Cummings et al., 1995; Kohrt et al., 1997). Overall, it would seem that any impact of PA may be more likely to be targeted to bone sites close to joints responsible for postural balance and ambulation as these experience regular absorption of GRFs to induce structural increases (Cummings et al., 1995; Kohrt et al., 1997). It is also interesting to note that it is possible that the significant negative associations between LIPA (∼12% of waking hours) and bone health may be a reflection of the overall low engagement in PA for this age group, hence any other lifestyle factors would have been likely to override its effects.

Our findings are also consistent with a report by others (Humphries et al., 1999), who concluded that the loss of bone may be of greater magnitude when compared to its formation during older age, more so in the women. Thus engagement in MVPA and LIPA may not be enough to prevent changes in bone metabolism solely; less time spent being sedentary may also be needed. Alternatively, findings may be attributed to the possibility that these older participants exhibited a number of other factors deleterious to bone formation and or maintenance. These could range, as often is the case with normal aging, from poor calcium retention, Vitamin D deficiency, or impaired parathyroid hormone secretion (Riggs et al., 1982; Cavanaugh and Cann, 1988; Humphries et al., 1999; Manolagas, 2000; Min et al., 2000; Chastin et al., 2014), and/or body composition/sub-optimal food intake (Tomlinson et al., 2019). Thus, any MVPA and LIPA in the habitual lifestyle of our female cohort in particular, was simply not sufficient to overcome these other factors. The fact that MVPA had a negative association with bone health in two cases in women (ribs BMD and total BMD), is also thought provoking. It could be that the MVPA-induced micro-damage may be slightly larger than the regeneration resulting in overall bone loss. Similarly, it is also highly likely that the activities of our participants did not reach the impact (acceleration > 4.2 g) or speed (10 km/h) purported as threshold needed to achieve sufficient stimulus for bone formation (Deere et al., 2012). Indeed, it has been shown that while master sprinters had a larger BMD than age-matched non-athletes, no such benefit was seen in even endurance master athletes (Piasecki et al., 2018). Also in line with the idea that a threshold of acceleration is required, accelerometer data in a sample of master athletes (Deere et al., 2016) and highly active postmenopausal women (Hannam et al., 2017) is shown to exhibit higher *Y*-axis peak accelerations in those compared with generally sedentary sex- and age-matched controls.

### Sedentary Behavior and Bone Health

In some cases, bone health was positively associated with the number of breaks in SB, but only within the cohort of men. Findings concur with previous studies whereby immobilization of the ambulatory limbs induced hormonal responses responsible for disruption of calcium metabolism necessary for bone formation (Kim et al., 2003, 2010; Smith et al., 2003; Zwart et al., 2009). Additionally, as biochemical markers of resorption (NTx, urinary calcium) are reported to elevate significantly after as few as 6 days of bed rest in a young healthy cohort; the assumption could be made that amongst a consistently sedentary, older cohort, these endocrine markers could also exist and be emphasized. Future studies should aim to collect serum and/or urine sample to describe any link between endocrine bone factors and SB. Indeed the hypothesis would be that where habitual loading is low, and hence the skeletal system is exposed to sub-optimal stress and strain, this would lead to less stimulation of bone formation and hence, in a shift from formation to resorption.

In contrast, high sleep time was seen to be detrimental toward BMD, with a negative correlation being established with several BMD sites, in the men but not in women. At the morphologic level, previous bed rest studies suggested that the hypoactivity-induced decreased BMD in men is accompanied by reductions in cortical area and cortical thickness, but increases in periosteal perimeter and trabecular area (Belavy et al., 2011).

It was surprising that high numbers of prolonged SB bouts were associated with better bone health. This may be partially explained by the BMI of these older participants being predominantly in the ‘overweight category.’ This has previously been reported to contribute to a higher BMD (Nordman et al., 2018). Increased loading, associated with the higher BMI, onto the skeleton is not necessarily the only route for this effect (Andersen et al., 2014).

In women, a higher number of short SB bouts and elevated total sedentary time (>60% of waking hours) were associated with a larger BMD. It remains unclear why the ribs region are particularly sensitive to disuse (Zerwekh et al., 1998). Indeed our conjecture is in line with the variation in the single Mechanostats setting, which favors the existence of different bone loading thresholds for different populations and bone sites (Skerry, 2006).

While at first glance these data may seem at odds with the benefits of loading for BMD (Frost, 1987a,b) it should be noted that the rib BMD in women was also positively related to the total weekly duration in PA. These apparently conflicting associations can be reconciled when one considers that a larger number of short SB bouts must imply a more frequent interruption of SB and hence a higher total PA. This and the fact that PA requires enhanced ventilation then results in enhanced loading of the ribs by the respiratory muscles and hence explain the positive relationship of rib BMD with both weekly PA duration and the number of short SB bouts. This then suggests that, at least in women, the rib BMD is positively influenced by PA. Alternatively, we would propose that a forward stooped posture commonly adopted by older persons, whilst walking and/or sitting, may be the cause for this regional effect. Indeed biomechanically speaking, the trunk region is kyphotic, and forward stooping would accentuate this curvature. A forward stoop would increase (forward) shear forces between thoracic vertebrae, and thus place additional stress/strain on the bone structures including the ribs, in this hyper-kyphotic position including para-spinal muscles and ligaments, thereby increasing the forces acting on the vertebrae (i.e., at their attachments).

### Study Limitations

A limitation for the present study was the lack of inclusion of detailed dietary parameters. Indeed while the DEXA scanning procedure includes a questionnaire on habitual dairy products intake, smoking habits and alcohol consumption, the details are not sufficiently refined (given these are self-reported data) to reliably include in the regressions. Precise data on a number of other factors that influence bone turnover would be ideal, including vitamin C and vitamin D intake, years post-menopause, family history (Riggs et al., 1982; Cummings et al., 1995; Chastin et al., 2014), and macronutrients diet composition and caloric intake (Tomlinson et al., 2019). Thus, future studies should take these into account in order to increase the granularity of our understanding of the unique impact of PB on bone health.

The paucity of positive associations between PA and bone site BMD, may be linked to a threshold of PA to affect bone. As we have discussed in the text above, it is possible that in their daily activities, this older age cohort (in carrying out PA at self-selected PA intensities and frequencies) may have self-selected activities inadequate to reach a key physiological threshold required to promote bone formation (Lanyon, 1992; Kohrt et al., 1997; Humphries et al., 1999). In addition, we note that running such a large number of correlations potentially increased type II errors, which was somewhat mitigated by looking at each sex separately and not overlapping our hypotheses. To build on our present work, we recommend that a future study with a larger sample size (including 15 participants for each PB outcome), utilizes a multiple linear/temporal substitution regression approach and estimates the power of such regressions based on the attained explained variance.

Last, we utilized one current week of PB and inferred this was a reflection of the long-medium term pattern, and this may not necessarily be true. However, to make the PB data as much as possible representative for the usual PB (1) we asked the participants to continue their daily life as usual and (2) included both weekdays and weekends that typically differ in PB even in retirees (McCormack et al., 2010). In future studies, PB may be monitored at two time points, separated by at least 6 months, to also adjust for potential seasonal variations in PB.

## Conclusion

In this sample of community-dwelling elders, PB is clearly able to distinguish one clinical sub-group from another. This is evidenced through bivariate correlations as well as group comparisons of overall PB (Z_PB_-scores). Indeed the latter is an approach which is part of the strength of the current study, providing as it does, both a visual and a quantitative representation of the overall PB pattern differences between samples (in our case bone health groups). What is also clear, is the sex specificity of these modulations. In fact, the Mechanostat theory (Frost, 1987a,b) does not apply indiscriminately: it is not necessarily where we see more loading that we may infer a healthier bone profile.

## Ethics Statement

Participants were recruited by word-of-mouth from a number of national organizations and local clubs (including the University of the Third Age (U3A), Rotary, Age United Kingdom, local golf clubs). Hundred and twelve adults (men, *N* = 51/women, *N* = 61) volunteered to participate in this study. All were aged between 57 and 89 years (average ± SD = males 73.6 ± 6.2 years, females 71.6 ± 6.4 years) and were of differing self-reported PA status. Full ethical approval was received through the Manchester Metropolitan University Ethics Committee prior to experimentation, and informed written consent was obtained from each participant.

## Author Contributions

GO-P, CM, and HD designed the research. JW and DR conducted the research. JW, DR, CD, and GO-P analyzed the data. CD, GO-P, and HD wrote the manuscript and this was reviewed by all co-authors. GO-P has primary responsibility for final content. All authors read and approved the final manuscript.

## Conflict of Interest Statement

The authors declare that the research was conducted in the absence of any commercial or financial relationships that could be construed as a potential conflict of interest.
